# Prospects of Inhaled Phage Therapy for Combatting Pulmonary Infections

**DOI:** 10.3389/fcimb.2021.758392

**Published:** 2021-12-06

**Authors:** Xiang Wang, Zuozhou Xie, Jinhong Zhao, Zhenghua Zhu, Chen Yang, Yi Liu

**Affiliations:** Department of Pulmonary and Critical Care Medicine, The Second People’s Hospital of Kunming, Kunming, China

**Keywords:** inhaled phage therapy, nebulizer, pulmonary infection, antimicrobial resistance, multi-drug resistance

## Abstract

With respiratory infections accounting for significant morbidity and mortality, the issue of antibiotic resistance has added to the gravity of the situation. Treatment of pulmonary infections (bacterial pneumonia, cystic fibrosis-associated bacterial infections, tuberculosis) is more challenging with the involvement of multi-drug resistant bacterial strains, which act as etiological agents. Furthermore, with the dearth of new antibiotics available and old antibiotics losing efficacy, it is prudent to switch to non-antibiotic approaches to fight this battle. Phage therapy represents one such approach that has proven effective against a range of bacterial pathogens including drug resistant strains. Inhaled phage therapy encompasses the use of stable phage preparations given *via* aerosol delivery. This therapy can be used as an adjunct treatment option in both prophylactic and therapeutic modes. In the present review, we first highlight the role and action of phages against pulmonary pathogens, followed by delineating the different methods of delivery of inhaled phage therapy with evidence of success. The review aims to focus on recent advances and developments in improving the final success and outcome of pulmonary phage therapy. It details the use of electrospray for targeted delivery, advances in nebulization techniques, individualized controlled inhalation with software control, and liposome-encapsulated nebulized phages to take pulmonary phage delivery to the next level. The review expands knowledge on the pulmonary delivery of phages and the advances that have been made for improved outcomes in the treatment of respiratory infections.

## 1 Introduction

Respiratory tract infections (RTIs) represent a leading cause of suffering and death worldwide. Respiratory diseases are the third major cause of mortality and sickness globally and account for more than 10% of all disability-adjusted life-years (DALYs) (GBD, 2016; Bodier-Montagutelli et al., 2017; WHO-Global Respiratory Burden, 2017). According to the World Lung Foundation’s Acute Respiratory Infections Atlas, acute RTIs cause more than four million deaths each year. A range of pulmonary infections develop into life-threatening and difficult to treat conditions with many leading to chronic conditions. Pneumonia is one such complication, which accounts for the highest number of juvenile deaths. As per data, a total of 9 million children under 5 years die annually, with pneumonia the leading killer among patients (WHO Pneumonia factsheet, 2019). Moreover, nosocomial cases of ventilator-associated pneumonia (VAP) refractory to traditional antibiotics are on the rise. This intensive care unit (ICU) acquired infection has a high incidence rate, ranging from 5%-40% in patients with mortality as high as 50% (Forel et al., 2012; Papazian et al., 2020).

Deaths due to tuberculosis (TB) are another similar eye-opener. Annually, a total of 1.4 million people die from TB in 2019, making it the leading cause of death from a single infectious agent, ranking above HIV/AIDS as declared by WHO (Global tuberculosis report WHO, 2018). Although TB is fully curable, the treatment is complicated due to the development of multi-drug resistance TB (MDR-TB) (Seung et al., 2015). The condition is still worse in the developing and third world nations and more than two-thirds of the active cases of TB reported globally come from these countries (WHO, Tuberculosis Factsheet, 2021).

Many other pulmonary conditions such as chronic obstructive pulmonary disease (COPD) and cystic fibrosis (CF) are easily associated and complicated with opportunistic bacterial pathogens (e.g *Pseudomonas aeruginosa* and *Burkholderia cepacia* complex-BCC) making the treatment and management a challenge adding significant financial burden, with poor clinical success achieved. It has been reported that the total healthcare cost for treating CF patients ranges to as high as 50,000 USD per patient per year and this is largely due to repeated hospital stays and treatment costs to manage the bacterial infections associated with such conditions (Sansgiry et al., 2012; Angelis et al., 2015; Trend et al., 2017).

This scenario is made worse by the decline in the effectiveness of current antibiotic therapies due to the rapid spread of resistant bacterial infections, which do not respond to traditional treatment protocols. The spread of antimicrobial resistance (AMR) is a global emergency and is very much prevalent and emerging in the pulmonary setting. Nosocomial outbreaks caused by resistant bacterial strains have been increasingly reported worldwide, creating significant therapeutic challenges for the treatment of lung infections (Rice, 2010; Santajit and Indrawattana, 2016). Moreover, there exists a dearth of new alternatives and with the present attrition rate in new antibiotic molecule developments by pharma companies, the situation is not encouraging, posing a serious threat and indicating that we are entering a post-antibiotic era (Nelson, 2003; Gupta and Nayak, 2014). This emphasizes the urgent necessity to promote and explore alternative approaches other than antibiotics that can be used either singly or better as an adjunct therapy for improved clinical outcomes and reduced mortality rates.

Among the approaches that are worth exploring is ‘Phage Therapy’. Phage therapy or the use of phages against bacterial pathogens is not a new concept. There has been a renewed interest in this field in recent years due to the rising resistance menace. Phage therapy represents a safe yet potent antibacterial strategy, with numerous reviews and data supporting and strongly advocating its further use (Carlton, 1999; Weber-Dabrowska et al., 2000; Sulakvelidze et al., 2001; Capparelli et al., 2007; Chanishvili, 2012). However, major roadblocks and challenges in this field need to be investigated.

The present review discusses the prospects of pulmonary phage therapy and its efficacy in treating various RTIs considering antimicrobial resistance. It later details the major challenges and new advances made in delivering phages to the infection site for improved outcome and availability, with major recent studies supporting these developments. This review aims to give a complete insight into the latest developments in inhaled phage therapy and how we can address the gaps and further improve it, which is essential for the clinical success of this new era of treatment.

## 2 Inhaled Phage Therapy: A New Era of Therapeutics

Phage therapy represents the use of obligatory lytic phages to kill specific host bacteria. The issue of growing multidrug resistance represents a potent and safe alternative. Phage therapy has shown both preclinical and clinical efficacy against a range of bacterial pathogens and data suggests that it also works well against pulmonary pathogens (Soothill, 1992; Biswas et al., 2002; Mathur et al., 2003; Wang et al., 2006; Watanabe et al., 2007; Chang et al., 2018; Chhibber et al., 2008). Inhaled phage therapy has long been used and is still in use in Eastern European countries i.e Georgia, Russia, and Poland. Studies focused on inhaled phage therapy in humans executed in these countries have been reviewed and compiled by Abedon (2015). There have been studies dating as early as 1936 wherein phages have been mostly delivered through inhalation route against a range of pulmonary pathogens such as *E. coli*, *Klebsiella*, Streptococci, Staphylococci, Pseudomonas, and the results of many studies have shown efficacy as high as 80-100% although some studies resulted in treatment failure owing to a lack of a better understanding of phage specificity, quality control, and stability issues (Hoe et al., 2013; Abedon, 2015; Chang et al., 2018). Modern phage therapy has come a long way and improved inhalation and aerosolization techniques have also helped to fill these gaps in knowledge about pulmonary phage therapy.

### 2.1 Major Pulmonary Pathogens: An Overview

VAP is a major nosocomial infection in which the causative agents can form biofilms on the surface of endotracheal tubes. These biofilms are mostly polymicrobial, with multiple organisms isolated (Hall-Stoodley and Stoodley, 2009; Rodrigues et al., 2017). This may not just include members of the oral flora (e.g., *Streptococcus and Prevotella* species), but also ESKAPE organisms (*Enterococcus faecium*, *Staphylococcus aureus*, *Klebsiella pneumoniae*, *Acinetobacter baumannii*, *P. aeruginosa*, *Enterobacter* spp) (Penes et al., 2017; Vadivoo and Usha, 2018). A hallmark of biofilms is the inherent resistance to killing and recalcitrance to antimicrobials and immune attack, outer environmental stress, enabling them to survive well within the body, leading to difficulty to treat chronic and relapsing infections (Romling and Balsalobre, 2012; Sharma et al., 2019). Biofilm cells can survive 100 to 1,000 higher concentrations of antimicrobials and biocides than planktonic cells (Gilbert et al., 2002; Høiby et al., 2010). Methicillin-resistant *S. aureus* (MRSA) is the second most frequently isolated pathogen from patients who die from HAP and is commonly associated with many cases of ICU acquired VAP (Rubinstein et al., 2008; Jean et al., 2020). It secretes a range of virulence factors, toxins, and biofilm-promoting adhesins that favor its colonization on medical devices and catheter tubings. Similarly, a major chronic lung disease i.e Cystic Fibrosis (CF) has gained much attention as its management and outcome are always complicated by persistent bacterial infections of the airways and destructive lung inflammation (Gibson et al., 2003; Pragman et al., 2016). With *P. aeruginosa* as the major pathogen isolated in most CF patients, this bacterium poses the greatest challenge, with treatment failures owing to its potent biofilm-forming ability and recalcitrant nature (Burns et al., 2001; Folkesson et al., 2012; Malhotra et al., 2019). Biofilm-like aggregates are commonly seen within the sputum in CF airways and such biofilms do not respond to courses of conventional antibiotics. Moreover, *P. aeruginosa* exhibits significant changes in gene expression, with up-regulation of exopolysaccharide production, excessive alginate production, and activated secretion of various quorum sensing molecules, multiple mechanisms of antibiotic resistance e.g overexpression of efflux pumps and beta-lactamases which further aid in the formation and survival of bacteria within the biofilm shelters (Lister et al., 2009; Harmsen et al., 2010; Poole, 2011). Another major pulmonary pathogen is the *B. cepacian* complex (Bcc) which consists of 18 closely related species that have been known to persist in the airways of people with CF although they are less common colonizers than *P. aeruginosa* (Lipuma, 2005; Drevinek and Mahenthiralingam, 2010; Kenna et al., 2017). These Bcc bacteria are associated with worst prognosis, high rates of morbidity and mortality amongst sufferers (Jones et al., 2004; Sousa et al., 2011; Hassan et al., 2019), owing to bacteria-induced acute-onset lung deterioration with associated septic bacteremia, termed ‘cepacia syndrome’. Apart from this, most members of this complex exhibit multidrug resistance and can form biofilms while evading immune attack (Tomlin et al., 2001; Mahenthiralingam et al., 2005; Van Acker et al., 2013).

Besides bacteria, one of the major pulmonary pathogens accounting for a large number of deaths annually showing a high degree of multidrug resistance is *Mycobacterium tuberculosis* (Mtb). The success of this intracellular pathogen is due to the ability of Mtb to remain hidden from the immune system, associated with relapse or frequent recurrence of active TB, which is often seen in patients despite anti-TB treatment (McCune et al., 1966; Jasmer et al., 2004; Ackart et al., 2014). Whether Mtb forms biofilms during infection remains unknown but notably, Mtb has a natural tendency to adhere to surfaces and forms cords in the culture medium and this cording behavior is associated with virulence and pathogenicity of Mtb (Esteban and García-Coca, 2018; Chakraborty et al., 2021). There have been recent studies reporting the formation of biofilm-like aggregates by this bacteria and the role of glycolipids, shorter-chain free mycolic acids, GroE-1 chaperone in the formation of these biofilms that play a detrimental role in causing caseous necrosis and cavity formation in lung tissue and treatment failures (Ojha et al., 2008; Sambhadan et al., 2013; Trivedi et al., 2016; Esteban and Garcia-Coca, 2018).

### 2.2 Phage Against Pulmonary Pathogens: Attack at Multiple Fronts

With this scenario, an ideal agent attacking the respiratory pathogens needs to exhibit remarkable anti-biofilm ability and phages completely fit into this class. Phage therapy works on multiple fronts to combat the course of pulmonary bacterial infections. Lytic phages work as killing machines. Lytic phages first bind to their target bacterium through specific receptors, injecting the genetic material and later taking over the host machinery for progeny phage production. The phage progeny are released from the host *via* cell lysis and the cycle restarts for many such rounds, leading to secondary infection. This property of self-replication or auto-doing enables phage titer build-up, which is essential for containment of the bacterial population.

Besides this conventional mode of killing, phages also exhibit anti-biofilm activity (Azeredo and Sutherland, 2008; Sillankorva and Azeredo, 2014; Morris et al., 2019). This is particularly important as biofilms play an important role in many pulmonary infections (Pintucci et al., 2010; Boisvert et al., 2016). They work on two fronts i.e they are capable of both preventing the onset and initiation of biofilm formation as well as disruption of fully formed biofilms, which they do in multiple manners. The successful eradication of an established biofilm requires the chemical or drug to have the ability to penetrate the EPS matrix and then kill the biofilm-embedded cells. Phages possess both these properties (Fu et al., 2010; Harper et al., 2014). Phages are naturally equipped with virion-associated de-polymerases and endolysins, phage-borne enzymes released at the later stages of the phage replication cycle, which degrade bacterial peptidoglycan and help in bacterial cell lysis and phage progeny release. These phage enzymes play an important role in dissolving the tough outer biofilm matrix (Roach and Donovan, 2015; Maciejewska et al., 2018). This allows the phages and progeny population to penetrate the deeper areas within the biofilm and kill the host bacterium through their classical killing mechanism Although phage diffusion may be slow in such layers, phages still retain their ability to bind and lysis the metabolically dormant or the slow-growing persister cells (unlike antibiotics) through receptor-mediated binding and killing. Apart from this, the phage-encoded de-polymerase enzyme degrades the EPS matrix, which not only facilitates the entry of phages but also makes way for the antibiotic molecule to gain entry into the biofilm structure and reach bacterial cells, thus leading to augmentation through the clearance and treatment outcome in a combination or co-therapy approach (Bedi et al., 2009; Ryan et al., 2012; Akturk et al., 2019). This phage-antibiotic synergy has been reported in past studies wherein phage enables the augmentation of antibiotics, making them ideal for use in combination mode with different antibiotics. This approach also decreases the development of resistant mutants (Bedi et al., 2009; Ryan et al., 2012; Tagliaferri et al., 2019).

Another major mechanism through which phages help to ameliorate the course of pulmonary infection is their ability to modulate the immune system towards a more subtle state, discouraging tissue damage. Studies report that phages administered for therapeutic purposes are able to regulate and reduce the heightened levels of inflammation. Phages have been shown to down-regulate the TLR expression which is the key molecule that leads to activation of NF-κB, leading to cytokine production, cell infiltration, phagocytosis. Many studies indicate a decrease in pro-inflammatory cytokine levels post-treatment (TNF-α, IL-1, IL-8, MIP-1) (Górski et al., 2012; Pabary et al., 2015; Kaur et al., 2016; Zhang L. et al., 2018; Cafora et al., 2020). Phages lead to inhibition of excessive reactive oxygen free radical production and also induce the production of anti-inflammatory cytokines, maintaining homeostasis while limiting cell and tissue injury (Przerwa et al., 2006; Borysowski et al., 2010; Van Belleghem et al., 2017). Phage ISP specific for S. aureus phage showed induction of anti-inflammatory IL-1 receptor antagonist (IL-1RA) synthesis by human monocytes, thus leading to the repression of pro-inflammatory cytokines (Van Belleghem et al., 2017). Two-way cooperation exists between phages and the immune system called “Immunophage Synergy”, whereby both complement each other towards faster resolution of infections and minimal tissue damage, as seen in neutrophil-phage cooperation, reported by Roach et al. (2017) against *P. aeruginosa*. In a similar study, Cafora et al. (2020) showed the anti-inflammatory role of the phage cocktail in terms of reduction of pro-inflammatory markers in *P. aeruginosa* infection using the zebrafish model. Phage cocktail injections significantly reduced neutrophil migration and heightened pro-inflammatory cytokine levels highlighting the molecular interaction between phages and the cells of the vertebrate immune system in CF disease and the anti-inflammatory role of phages. Similar animal studies have shown the anti-inflammatory ability of phages in downregulating the exaggerated immune response by decreasing the levels of pro-inflammatory cytokines during the infection process against a range of pulmonary pathogens (Chhibber et al., 2008; Kumari et al., 2019). The immune-modulating ability of phages is a unique field and requires further investigation. Another important point worth mentioning is the fact that phages may themselves also evoke an immune response and thus phage preparation needs to ensure high purity (Boratyński et al., 2004; Liu et al., 2021) as it becomes an extremely important parameter in determining whether phage therapy will reduce or promote inflammation and antibody response.

**Figure 1:**
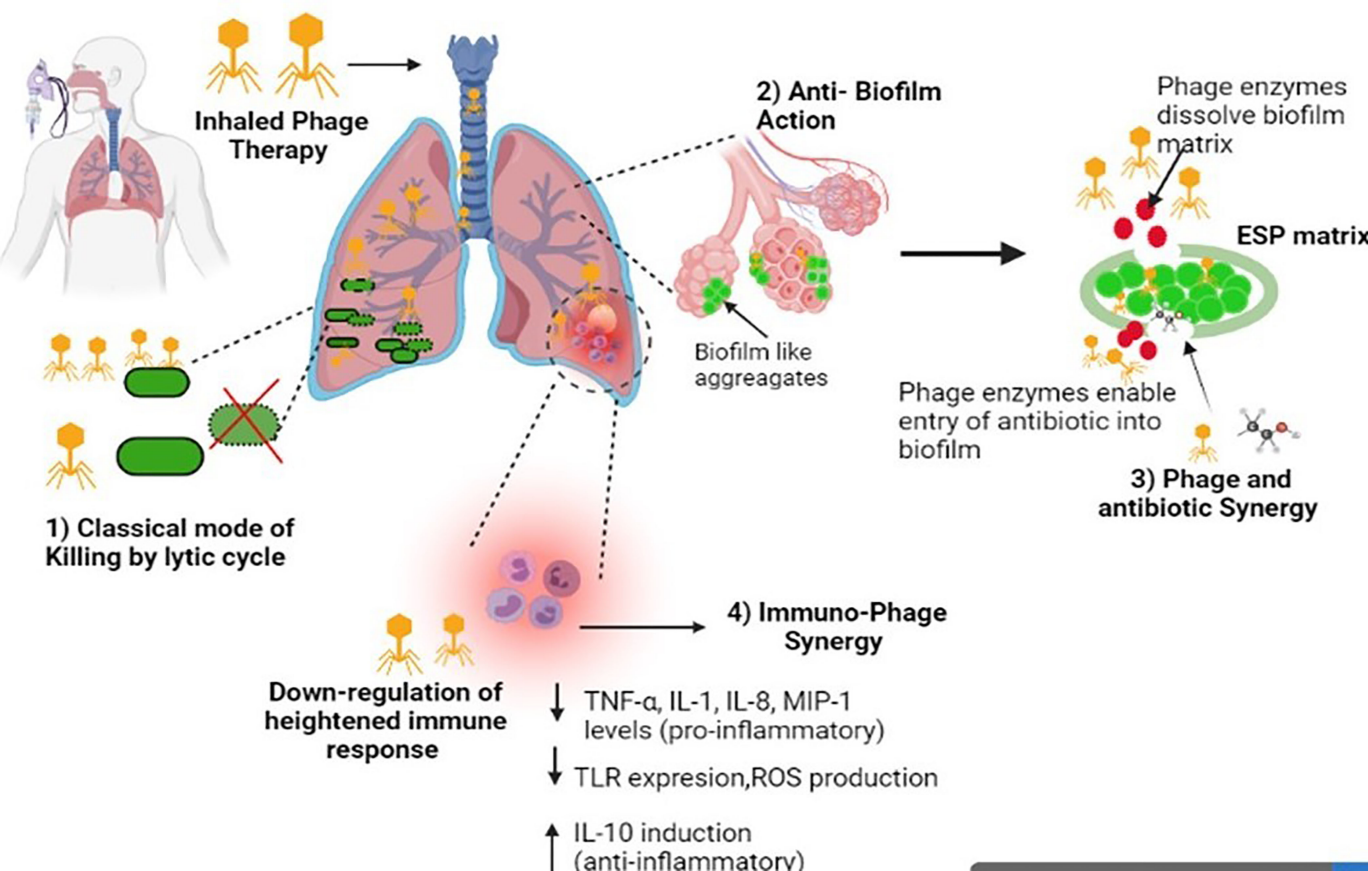
Diagrammatic illustration of the multiple mechanisms of phages against respiratory infections. Image created in Biorender.

Free phages have limited ability to enter the human eukaryotic cells and reach intracellular pathogens (Sulakvelidze et al., 2001; Drulis-Kawa et al., 2012; Doss et al., 2017). Despite this, a few studies indicate that phages can penetrate eukaryotic cells and attack the intracellular populations of pathogens by adopting a different strategy to invade the eukaryotic cells. Kaur et al. (2014) evaluated the intracellular killing potential of macrophages in the presence of free phage MR-5 as well as phage adsorbed onto host MRSA strain. Results depicted that free phage did not influence intracellular killing of engulfed *S. aureus* by macrophages while phage adsorbed onto its host bacterial cells showed a time-dependent and titer-dependent significant reduction in the number of viable intracellular cocci. This means that phages utilized host bacteria as a vehicle to shuttle inside the macrophage and kill the intracellular cocci. In another study, the use of a novel delivery Trojan Horse approach i.e *Mycobacterium smegmatis* as a carrier for delivering phages to the intracellular bacteria (Broxmeyer et al., 2002) was reported. Another strategy to deliver phages intracellularly is *via* the use of liposomes, which is detailed in later sections of this review. The multiple fronts at which phages attack respiratory bacterial pathogens and thus ameliorating the disease outcome are compiled in **Figure 1**.

### 2.3 Pulmonary Phage Delivery

Having studied the multiple mechanisms of phage therapy, we now focus on the different methods that have been adopted in the delivery of phages to pulmonary sites along with proof of data against the clinically relevant pathogens causing RTIs. In later sections, we focus on the challenges and advances made in this direction.

#### 2.3.1 Nebulizer Based Inhalation

Nebulization is the process of creating of converting liquid into a fine mist of active ingredient solution through the special nozzle. It is the first choice for delivering drugs through the pulmonary route, owing to its high efficiency and ability to deliver high volumes of the agent due to liquid-based preparations (Ali, 2010; Haddrell et al., 2014; Martin and Finlay, 2015).To deliver a drug by nebulization, the drug must first be dispersed in a liquid (usually aqueous) medium. After the application of a dispersing force (either a jet of gas or ultrasonic waves), the drug particles are contained within the aerosol droplets, which are then inhaled (Javadzadeh and Yaqoubi, 2017). There are many types of nebulization-based methods that use different mechanisms to produce aerosols. In jet nebulization, which worked on Venturi Principle, compressed air is passed through a narrow orifice with a force that leads to a pressure gradient, and this enables to draw/suck the drug suspension up into the feed tube. The liquid gets atomized into micron size droplets *via* viscosity-induced instability whereas the larger droplets are trapped and filtered by the baffle (Finlay, 2001; Hickey, 2004). The smaller droplets leave the nebulizer to reach the target sites, but the bigger droplets are returned to the reservoir for re-nebulization. In vibrating mesh nebulization, the generation of droplets occurs either by a piezoelectric crystal that vibrates at high frequency and then these vibrations are transferred to a transducer that pushes the liquid suspension upward and downward through a mesh plate to extrude the liquid and generate aerosol droplets or in active arrangement, the crystal directly vibrates the mesh plate (Vecellio, 2006; Ali, 2010). Ultrasonic nebulizers also work on the principle of converse piezoelectric effect, whereby the piezoelectric crystal vibrates high frequency acoustic energy waves (no mesh plate is required here), which leads to aerosol creation (Flament et al., 2001; Rau, 2002). The vibrations are transmitted to the drug solution *via* a buffer medium. Droplets are formed either by the breakup of surface capillaries or by the collapse of cavitation bubbles within the liquid. Ultrasonic nebulizers produce a more uniform particle size than jet nebulizers but are less widely used due to high pricing (Flament et al., 2001; Rau, 2002; Javadzadeh and Yaqoubi, 2017). To date, many drugs have been nebulized and given through this technique to reach the lungs and show their desired action. Phage-based nebulization has been reported by past studies using different types of nebulizers. However, one major disadvantage is that each of the nebulization processes is associated with the mechanical stress produced during the formation of high-frequency vibrations, passing of compressed air, heat stress during the generation of high-frequency acoustic energy, re-nebulization process, shear stress during impaction. These physical stresses can have potentially damaging effects on the phage structure leading to a reduction in phage viability and infectivity. A study by Turgeon et al. (2014) reported that aerosolization produced by nebulizers led to reduced infectivity of five tail-less phages while 1000-fold more genome copies were found in the nebulized product as compared with low PFU values suggesting that nebulizers damaged the structural integrity of phages required for infection. Air jet nebulizers and, to a lesser extent, vibrating mesh nebulizers, cause tail detachment leading to broken phages that are incapable of initiating the phage infection process. Another change in phage morphology is the production of phages with empty capsids and mesh-type nebulizers, which produce a higher fraction of such phages (Astudillo et al., 2018). The stress of liquid breakup, large hydrodynamic stress with high-speed nozzle, and impaction on primary and secondary baffles contribute to the damaging effect on phage structure leading to loss of tails and nucleic acid ejection (Carrigy et al., 2017; Leung et al., 2019). Hence, the selection of the nebulization mechanism that best suits a particular phage may vary, and thus detailed post-nebulization studies on phage titer and viability need to be carried out for each nebulization technique.

In a study, the effect of three types of nebulizers i.e air-jet, vibrating-mesh, and static-mesh nebulizers, on the structural stability of a *Myoviridae* phage, PEV44, active against *P. aeruginosa* was visualized by TEM. Results indicated that the fraction of “broken” phages (capsid separated from the tail) was significantly increased after the process of nebulization and maximum was obtained with the air-jet nebulizer (83%) while with mesh- nebulizers it was 50 - 60% (Astudillo et al., 2018). In a study by Leung et al. (2019), the team examined the susceptibility of phage from different morphological classes against the same type of jet-nebulizer with transmission electron microscopy (TEM). Results showed that phage degradation was closely associated with phage tail length. Phage PEV2, a podovirus characterized by a short, stubby, non-contractile tail showed negligible effect with a similar fraction of intact and viable phages (with filled capsids) post-nebulization in parent stock solution. However, in the case of PEV40 (a myovirus characterized by a long, straight, contractile tail) and D29 (a siphovirus characterized by a long, flexible, non-contractile tail), the fraction of intact structures seen after nebulization were considerably reduced from 50% to ∼27% for PEV40 and from 15% to ∼2% for D29 respectively. This suggested that the long-tailed phages were highly susceptible to the stress of the jet nebulization process compared to phages with stubby short tails and for these long tail phages, the tail detachment was the most common type of damage. This damaging effect can be minimized by the presence of organic fluid in the nebulization buffer, which exhibits a protecting effect on phage morphology in the case of some phages (e.g phages PR772 and Φ6) throughout the aerosolization, as observed by Turgeon et al. (2014).

In another *in vitro* study, Golshahi et al. (2008) investigated the suitability of respiratory phage administration by nebulization method against *B. cepacia* complex. Phage KS4-M at titers with mean value (standard deviation) of 2.15x10^8^ (1.63 x 10^8^) PFU/ml was aerosolized with Pari LC star and eFlow nebulizers and the breathing pattern of an adult was simulated using a pulmonary waveform generator. The size distributions of the nebulized aerosol and the overall efficiency of nebulizer delivery were measured. The aerosol was collected on low resistance filters (at the exit of the nebulizer mouthpiece) and the phage counts were determined termed as the “phage inhaled count.” The data was then used in a mathematical lung deposition model to predict the regional deposition of phages in the lung. Results based on a mathematical model suggested that LCstar and eFlow both appear suitable for BCC phage therapy with good inhaled and deposition titers. Phages (post nebulization) showed high phage inhaled counts i.e 1.06 x10^8^ and 1.15x10^8^ PFU for the LC star and eFlow, respectively, more than that effective dose required in mice (10^7^ PFU). The alveolar deposition predicted was also high i.e 3.02 x 107 PFU (LCstar) and 2.96 x 107 PFU (eFlow). Thus, respiratory phage delivery *via* nebulization has potential in resolving BCC infection in cystic fibrosis patients, and further *in vivo* work in this direction is warranted.

Other factors such as relative humidity and temperature may also effect the final selection of the most suitable method of nebulization. Liu et al. (2012) showed that relative humidity has a strong effect on the cultivability of the mycobacteriophage D29 and it was best seen in a low-humidity condition (25%) than medium to high (55-85%). They also assessed the aerosolization (by collision jet nebulizer) process in the presence of three different spray liquids—deionized water, phosphate buffered saline (PBS), and normal saline. The results indicated that significantly more D29 aerosol particles were generated when the spray fluid was deionized water than PBS or normal saline. Phage particles generated by deionized water retained better viability (30-300 fold more) than those prepared in PBS and normal saline, hinting toward the fact that both high salt concentration and high ionic strength have negative effects on the bioaerosol generation process. The aerosols generated with deionized water had a median mass aerodynamic diameter (MMAD) of 2.4 μm, which is well within the range (1-3 μm) required for reaching and depositing within deeper lung pockets (Thomas, 2013).

The above studies indicate that for morphologically stable phages (that can tolerate the sheer stress produced), both jet and vibrating mesh nebulization represent ideal choices for aerosolized delivery, achieving high rates of deep lung deposition that are essential for the effective resolution of respiratory infection. However, this may not be the case for all phages, and multiple factors such as phage-type, its sensitivity, temperature and humidity conditions, method of nebulization all impact the outcome. These need to be optimized for the best phage-inhalation technique combination and to get the least phage titer loss and highest lung delivery. Some major efficacy studies using different nebulization methods are outlined in **Table 1**.

**Table 1:** Treatment outcomes of recent *in vivo* efficacy studies and clinical case studies in which different inhalation delivery methods were used in animal models.

Delivered as:	Bacteria	Phage involved	Study highlights	Main Findings	Reference
Liquid Aerosol (LC-star jet nebulizer)	*B. cenocepacia* K56-2 and C6433	Phages KS4-M, KS5, and KS12 against *B. cenocepacia* K56-2 Phages DC1 and KS14 against *B. cenocepacia* C6433	Experimental *B. cenocepacia* (BCC) respiratory infections were established in mice and post-infection, animals received treatment with one of five bacteriophages specific to this bacterial species, administered as an aerosol or intraperitoneal injection. Bacterial and bacteriophage titers were determined in the animals’ lungs after 2 days	BCC-infected mice treated with aerosolized phage treatments showed a significant decline in bacterial load in affected lung tissue Phage KS12 given at an MOI of 131 produced a 2.5-log mean reduction in *B. cenocepacia* K56-2 counts two days post-infection. Phage KS5 given at MOI of 32 produced a 3-log mean reduction in *B. cenocepacia* K56-2 in the lungs one day post-treatment and further high reduction of 4-log were observed 3 days post-treatment. Nebulization is a more effective way in delivering phage particles to the lung than other methods.	Semler et al., 2014
Liquid Aerosol (Penn-century aerosolizer, Collison 6-jet, and Spinning top aerosol nebulizers)	*M. tuberculosis*	D29 mycobacteriophage	Deposition and distribution of aerosolized phage D29 particles in naive BALB/C mice were studied. Phage D29 aerosols were given to animals by endotracheal route using Penn-century aerosolizer; Collison 6-jet and spinning top aerosol nebulizers (STAG) and also compared with nose only route. Post-exposure, the deposited amounts of phage D29 particles in respiratory tracts and deposition efficiencies were calculated.	10% of D29 phage could reach the lung of mice after nebulization and complete phage elimination was noted in 72 h, whereas only 0.1% of the phage could reach the lung by IP injection and no phage was detected after 12 h. Also, no inflammation was observed in the lungs of mice receiving phage aerosols as per the BALF analysis Aerosol delivery of phage D29 is an effective way of treating pulmonary infections caused by *M. tuberculosis*.	Liu et al., 2016
Powder Aerosol (DPI)	*P. aeruginosa*	Phage PEV20	Phage PEV20 spray dried inhalable powder with lactose and leucine produced. Multidrug-resistant (MDR) strain *P. aeruginosa* FADDI-PA001 was established in a mouse lung infection model. At 2 h after the bacterial challenge, mice were treated with 2 mg of phage dry powder using a dry-powder insufflator.	Bacterial load got reduced by ~0.5 log in mice received phage *via* ip route while 2-log bacterial reduction was observed in the group treated with inhaled phage. Nebulization is a more effective way in delivering phage particles to the lung than intranasal instillation	Chang et al., 2018
Liquid Aerosol (Vibrating mesh nebulizer)	*M. tuberculosis*	D29 phage	Prophylactic pulmonary delivery of active aerosolized phage D29 was studied in female C57BL/6 mice. An average phage conc. of 1 PFU/alveolus was delivered *via* nose-only inhalation device using a dose simulation technique and then adapted for use with vibrating mesh nebulizer. Post 30 min, mice were given either a low dose (~50-100 CFU) or an ultra-low dose (~5-10 CFU), of bacteria aerosols. Bacterial burden of Mtb was evaluated 24 hours and 21 days post-challenge for the low dose model and at 24 hours for the ultra-low dose model.	A prophylactic effect was observed with phage aerosol pre-treatment significantly decreasing *M. tuberculosis* burden in mouse lungs 24 hours and 3 weeks post-challenge. This represents a valuable prophylactic approach for the healthcare professional and staff that are at high risk of exposure to *M. tuberculosis*.	Carrigy et al., 2019a; Carrigy et al., 2019b
Spray dried Powder Aerosol (DPI)	*P. aeruginosa*	Phage PEV20 and ciprofloxacin	Inhalable powder of Pseudomonas phage PEV20 with ciprofloxacin by co-spray drying was developed. Mouse model of neutropenic mouse model of acute lung infection was established. Post-infection, different mice groups were given spray-dried single PEV20 (10^6^ PFU/mg), single ciprofloxacin (0.33 mg/mg), or combined PEV20-ciprofloxacin treatment using a dry powder insufflator.	Significant reduction in lung bacterial load (as high as by 5.9 log_10_) was obtained with PEV20 and ciprofloxacin combination powder along with reduced inflammation in the lung unlike when either phage or ciprofloxacin were given singly.	Lin et al., 2021
pMDI	*P. aeruginosa*	FKZ/D3 and KS4-M phages	Aqueous FKZ/D3 and KS4-M phage solutions were formulated in a reverse emulsion with Tyloxapol surfactant and filled into hydrofluoroalkane 134a pMDI canisters (50-µl metering valve). The canisters were shaken well, and five actuations were collected. The phage titer loss post-actuation was measured. Storage stability was not tested.	Phage titer loss was less than one log PFU thus maintaining good viability of both the phages. Phage delivery from a pMDI showed an acceptable titer loss for the two myoviridae phages post actuation.	Hoe et al., 2014
Liquid Aerosol (Modified Vibrating mesh nebulizer)	Methicillin-resistant *S. aureus* (MRSA) clinical isolate AW7	Phage cocktail of four phages (2003, 2002, 3A, and phage K)	Male Wistar rats were divided into different groups and ventilated for four hours and after ventilation, rats were inoculated *via* the endotracheal tube with MRSA then extubated. Different animal groups received: aerophages; intravenous (IV) phages; a combination of IV and aerophages; a combination of IV linezolid and aerophages. Aerophages were delivered using a modified vibrating mesh aerosol drug delivery system (1.5 × 10^10^ PFU] The primary outcome was survival at 96 hours.	The inhaled phage cocktails given with IV, and delivered phages given alone could each rescue 50% of test animals from death due to MRSA pneumonia. In combination mode of aerophages and IV phages, 91% of animals were saved from death. But when aerophages were given along with linezolid no synergistic effect was seen and there was a 55% survival. Aerosolized phage therapy showed potential for the treatment of MRSA pneumonia.	Prazak et al., 2020
**Case studies** of Pulmonary Phage therapy in humans					
Liquid Aerosol (Collision-jet nebulizer)	MDR- *Achromobacter xylosoxidans*	Cocktail of two *Achromobacter* phages (Siphoviridae) prepared at Eliava Institute, Tbilisi)	A case of 17 year old female with cystic fibrosis and chronic infection with *A. xylosoxidans* (starting at age of 12) not responding to many rounds of antibiotics. Phage was administered *via* inhalation using a compression nebulizer once daily (3x10^8^ PFU/ml) and phages were also given orally twice daily for 20 days. The same treatment course (inhaled plus oral) was repeated four times (at 1 month, 3 months, 6 months, and 12 months).	After the initial round of phage treatment, the patient’s conditions significantly improved, dyspnea resolved, and cough reduced. Her lung function measured as Forced expiratory volume (FEV1) increased from an initial 1.83 L (54%) to 1.88 L (62%) in 3 months post treatment. After the final treatment t round of *Achromobacter* phages, there was a significant improvement in lung function reaching to a final FEV1 value of 3.33 L (84%).	Hoyle et al., 2018
Liquid Aerosol (Vibrating mesh nebulizer)	Carbapenem-resistant *A. baumannii* (CRAB)	Personalized lytic pathogen-specific single-phage (Unnamed)	A case of an 88-year-old man already suffering from chronic obstructive pulmonary disease developed hospital acquired pneumonia (HAP) with carbapenem-resistant *A. baumannii* as the etiological agent. A personalized single-phage preparation was nebulized to the patient continuously for 16 days in combination with tigecycline and polymyxin E.	The treatment was well tolerated and resulted in clearance of the infection from patient’s lung with clinical improvement in lung function.	Tan et al., 2021
Liquid Aerosol (Vibrating mesh nebulizer)	*Achromobacter xylosoxidans*	Cocktail of three lytic phages ((JW Delta, JWT, and 2-1)- APC 1.1 And another cocktail mix (APC 2.1) with phage JWalpha was added to the above three phage cocktail.	A 12-year-old lung-transplanted cystic fibrosis patient with persistent lung infection with pandrug-resistant *A. xylosoxidans* Patient received two rounds of phage therapy. In first round 3 nebulizations/day of 5 mL (10^10^ PFU/ml) of APC 1.1 phage cocktail. In the second round, APC 2.1 was given (phage JWalpha added to the previous cocktail mix) and given. Initially, 30 mL of APC 2.1, tenfold diluted was instilled in each pulmonary lobe, and later on, discharge, continued phage nebulization at home: three times a day 5 mL of preparation for 14 days.	Clinical tolerance was perfect after each round of therapy with no observed side effects. However, the culture was positive with bronchoalveolar lavage (BAL) showing low densities of *A. xylosoxidans.* But, overall there was a constant improvement in the respiratory condition, and oxygen therapy was stopped. Low-grade counts of *A. xylosoxidans* (10^3^ CFU/ml) persisted for months and finally turned negative although it took almost 10-12 months. No re-colonization occurred more than two years after phage therapy was stopped.	Lebeaux et al., 2021

#### 2.3.2 Dry Powder Inhalation

Although nebulization is the preferred method for phage delivery, other areas of delivery *via* inhalation include solid phage formulations or dry powder inhalation-based methods. Since phage essentially consists of coat proteins, the protein-based formulations are better suited, as proteins tend to show higher stability in a dry state than in solution form (Cicerone et al., 2015) and hence dry powder formulation show enhanced shelf–life. This accounts for the main advantage favoring this method, which stems from the high degree of phage stability seen during the transport and storage period of such formulations, which is always preferred and required (Chang et al., 2018). Unlike the mechanical stresses associated with nebulizers (ultrasonic, vibrating mesh, or jet type), which may have a detrimental effect on phage morphology, physical stresses are not encountered during the preparation of dry powder aerosols. DPIs are breath-actuated, and the patient’s inhalation helps to disaggregate the powder into smaller particles (Geller, 2005). Apart from this, the ease of handling, fast delivery time, no need for electricity for operation, and no regular disinfection make it worth exploring as an ideal delivery platform (Zhou et al., 2014; Respaud et al., 2015).

There are primarily three main ways of producing phage-based dry powders for use which include a) spray drying (SD) b) freeze-drying (FD) and c) spray freeze drying (SFD). Briefly, SD is a single-step method for producing dry powders from liquid suspensions using a gaseous hot drying medium. This occurs in a phased manner whereby the liquid solution upon entering the atomizer gets broken into a spray of fine droplets followed by the droplets being ejected into the drying gas medium, allowing moisture vaporization to form dry particles and final particle collections (Mujumdar, 2007; Vehring, 2008). However, in the SD method, drying occurs when a continuous liquid film is converted into droplets followed by exposure to a hot, dry airflow. The increase in heat exchange area with a high-temperature difference enables to speed up the drying process (Moreira et al., 2021). However, the heat exchange process can have a direct damaging effect on thermosensitive phages affecting their viability. FD addresses this issue of preserving heat-labile components, as it involves a low-temperature dehydration method whereby the solvent (mostly water) is first frozen into ice and later removed by sublimation (direct transition from solid to vapor state) obtained under low pressures in a vacuum chamber (Schwegman et al., 2005; Liu, 2006). The long drying times, drying cycles, and the high vacuum used may cause additional damage, leading to a loss in phage titers during the lyophilization process itself (Lopez-Quiroga et al., 2012; Ishwarya et al., 2014). The more recent non-conventional SFD method uses a combination of a series of steps i.e droplet formation, freezing, and sublimation, producing uniquely powdered products. SFD is a unique drying technique, as it is a combination of both spray drying and freeze-drying. Furthermore, the unique aerodynamic qualities of the porous particles produced during SFD make it attractive for use in pulmonary delivery (Wang et al., 2006; Filkova et al., 2007). SFD has proven benefits with improved structural integrity, superior quality, and better shelf stability than existing drying techniques (Ishwarya et al., 2014; Fukushige et al., 2020).

Having been used for a long time, FD is a common method for reducing the dry powders of different drugs with high storage stability. One necessary parameter is the use of excipients for effective phage lyophilization and also for protecting their viability (Malenovská, 2014; Manohar and Ramesh, 2019). The type and concentration of excipients and stabilizing agents used need to be optimized. Using the traditional FD method, Puapermpoonsiri et al. (2009) developed inhalable dry powders of *S. aureus* and *P. aeruginosa* phages. The phage-loaded poly (lactic-co-glycolic acid) (PLGA) microspheres were first optimized and later lyophilized to form powders. This system although showed a desirable release profile i.e a burst release phase followed by a sustained release till 6h, but encapsulated phage got deactivated within 7 days either stored at 4°C or 22°C. Similarly, a study by Merabishvilli et al. (2013) focused on evaluating the choice of different stabilizers on *S. aureus* phage ISP free dried preparation over 37 months at 4°C. This study showed that sucrose and trehalose were the best-stabilizing additives, causing a decrease of only 1 log immediately after the lyophilization procedure with high stability over the test period. These sugars act as water substitutes and have a stabilizing effect on phage titers over the storage period.

As freeze-dried powders are not respirable, after their production an extra milling step is required to reduce the particle size to <5 μm, which is ideal for pulmonary delivery. However, this milling process may cause loss of phage due to the mechanical stress produced (Yan et al., 2021). Golshahi et al. (2011) prepared endotoxin-free lyophilized formulations of KS4-M and ΦKZ phages with 60% lactose and 40% lactoferrin as the selected cryo-protectant and stabilizers and then de-agglomerated in a mixer mill (without beads) to formulate respirable powders and aerosolized using an Aerolizer^®^ capsule inhaler. Post-lyophilization, there was a titer loss in the range of 1-2 log_10_ for both phages and the size of the phage powder was within the inhalable range (< 5 μm). The freeze-dried phage powders showed good stability with negligible titer reduction within 3 months when stored either at 4°C or 22 C in controlled relative humidity (RH of 21 ± 2%). *In vitro* aerosol testing showed that the phage titers collected downstream of the mouth throat were within the range of 10^6^-10^7^ PFU with a slight titer drop from capsule dose to respirable dose (titer loss of 1.2 log_10_ for KS4-M, and 0.84 log_10_ for ΦKZ phages), which was acceptable.

The SD method produces fine drug particles for pulmonary delivery as a single-step method and is less expensive than FD. SD method works well to maintain the stability and activity of phages and this work was initiated by Matinkhoo and co-workers (2011) produced dry powder inhalable formulation of bacteriophages KS4‐ M, KS14, and cocktails of phages ΦKZ/D3 and ΦKZ/D3/KS4‐M using a low‐temperature spray‐drying process due to thermal sensitivity of phages. In the formulation, trehalose was used to protect phage against dehydration, while leucine added helped to enhance the dispersibility of powders. The aerosol performance of the resulting dry powders was measured by determining their median mass aerodynamic diameter (MMAD). MMAD represents the aerodynamic diameter at which half of the aerosolized drug mass lies below the stated diameter. It is the average size of particles constituting the dose that reaches the impactor. Particles with an aerodynamic diameter of between 0.5 to 5 μm show a high probability of reaching and depositing in the lung and small ones can penetrate deeper lung tissues (Sheth et al., 2015). However, aerosol particles with a diameter larger than 5 μm tend to remain deposited in the throat or oropharyngeal cavity and fail to reach the lungs. In this study, the SD phage powders had an MMAD diameter of 2.5–2.8 µm suitable for pulmonary delivery of phages to reach the lungs. The actual phage dose reaching lungs released from a single actuation of the inhaler ranged from 10^7^ to 10^8^ PFU. According to past studies, this phage dose is likely to be effective at containing infection, with phage being effective at these PFU values (Wright et al., 2009; Morello et al., 2011).

Another important parameter that is crucial for determining drug efficacy in the case of pulmonary delivery is pulmonary deposition (highest dose fraction deposition in the lower airways i.e deep lung areas rather than lost in the oropharyngeal sphere), which not only depends on the inhalation device used but also on the ability of the dry powder to be dispersed in the air i.e powder dispersibility (Labiris and Dolovich, 2003; Newman, 2017). The fine particle fraction (FPF) represents the proportion of emitted particles that have a lower particle size than the diameter of the upper airway, which is fixed at 5 µm (Guo et al., 2013; Sibum et al., 2018). To enhance the FPF value, higher dispersibility is essential. This is a delicate process, as micron-sized particles are generally very cohesive and adhesive. The use of excipients needs to be optimized for each formulation. Amino acid, i.e leucine and trileucine, are often used as excipients to SD powders as enhancers of dispersibility and to provide moisture protection (Lechanteur and Evrard, 2020; Zhang Y. et al., 2018). SD powder is mostly amorphous and tends to gain moisture leading to agglomeration. These amino acids exhibit surface-active properties and form a hydrophobic shell that protects spray-dried particles from moisture (Matinkhoo et al., 2011; Mah et al., 2019). For example, with the addition of 20% (w/w) l-leucine to a range of formulations, there was a significant increase i.e 17.3–41.5% for FPFs (Momin et al., 2019; Stewart et al., 2019; Lu et al., 2020). Similarly, when 37.5% (w/w) leucine was added to a spray-dried formulation of budesonide, FPP values increased by 28% (Simková et al., 2020). Next, we have different sugars (lactose, mannitol, trehalose, sucrose), which act as a diluent and flow enhancer, improving aerosolization properties. Further sugars act as stabilizers and protect the active drug during drying and subsequent storage (Zhang Y. et al., 2018; Zillen et al., 2021). However, in the case of sugars, another important parameter to be investigated is the value of glass transition temperature (Tg) of the chosen sugars, which is the temperature at which an amorphous system changes from the brittle glassy state to a viscous rubbery state. Sugars with low Tg tend to crystallize easily, such as mannitol, which has a very low Tg value (Pyne et al., 2002), while Trehalose has a relatively high Tg i.e 106°C and hence is a suitable stabilizer, as it forms a glassy sugar matrix (Buitink et al., 2000).

In addition to these factors, the choice of excipients has a significant impact on maintaining phage stability and phage titers in the final formulation. Chang R. Y. K. et al. (2017) focused their study on evaluating the effect of excipients on the stabilization of spray-dried powders against anti-pseudomonal phages of different morphologies. Both podovirus and myovirus phages showed high stability with trehalose or lactose and leucine as excipients with a negligible loss of less than one log titer. Still, lactose showed superior phage protection over trehalose. Lactose has also been only approved by the FDA as a stabilizing excipient for use, while others may need more safety and regulatory approvals. On similar grounds, the same team then evaluated the storage stability of inhalable phage powders with lactose and leucine as excipients at 20°C/60% RH for 12 months. Results indicated that 90% lactose was able to maintain the viability of phage over the 12 months storage period while ∼1.2 log_10_ titer reduction was observed in formulations with less lactose. The spray-dried anti-pseudomonal phage powders were also shown to be non-toxic to lung alveolar macrophage and epithelial cells *in vitro* (Chang et al., 2019). Thus, leucine not only helps to minimize recrystallization of trehalose/lactose during the powder production process, preventing particle merging and enhancing powder flow but also showing a stabilizing effect on maintaining phage titers. Similarly, trileucine has also been shown to maintain high phage stability when used as excipients in phage-based formulations. In one such study, Carrigy and the team (Carrigy et al., 2019a, 2019b, 2020) evaluated the stability of engineered spray-dried microparticle based phage formulations of anti-Campylobacter bacteriophage CP30A. They produced amorphous spray-dried powder with excipient formulations containing trehalose and a high glass transition temperature amorphous shell former, either trileucine or pullulan. Results showed the high stability of phage titers with a combination of trileucine and trehalose, with titer reduction of only 0.6 ± 0.1 log_10_ (PFU/ml) over a 30 day period of storage. Such SD formulations can thus be safely transported a long-distance without the need for maintaining a cold chain system, thus cutting the cost by significant margins. Besides the choice of excipients, the temperature and relative humidity during the storage of SD preparations are equally crucial. Studies on RH show that formulations stored at high humidity conditions (RH > 50%) showed recrystallization of the amorphous content and hence SD powders need to be stored at low humidity conditions (RH ≤ 20%) (Vandenheuvel et al., 2014; Leung et al., 2016). It is also generally recommended to store phage drug powder at a temperature at least 50°C below the glass transition temperature (Tg) of the powders (Chang et al., 2020).

More recently, an SFD method of producing dry phage powders has been developed. This method shows enhanced structural integrity and stability over other drying methods. SFD yields particles of sizes and densities that show higher stability in the lungs and nasal mucosa (Vishali et al., 2019). SFD has also been shown to produce powders with particles larger and more porous than spray drying (Maa et al., 1999). Leung et al. (2016) compared both SD and SFD methods of procuring inhalable phage powders of *Pseudomonas* podoviridae phage, PEV2. Their results showed a loss of 2 log titers in the SFD method owing to the use of ultrasonic nozzle but the *in vitro* aerosol performance showed that the SFD powders showed significantly higher phage recovery (~80% phage recovery) compared with the SD counterparts (~20% phage recovery). This needs to be taken into consideration while using the SFD based method due to phage sensitivity to the mechanical stress. The frozen powders in SFD are also dried under vacuum pressure, adding to the long drying times (Shoyele and Cawthorne, 2006) that may cause more titer loss. While addressing this issue, Ly and their team (2019) studied a new technique of atmospheric spray freeze drying (ASFD) in developing a solid dry formulation of mycobacterium phage D29. In this process, phage D29 (in presence of varying concentrations of trehalose and mannitol) was sprayed and then frozen in a cold chamber followed by the passing of cold drying gas through the chamber resulting in the sublimation of ice forming a free-flowing powder. The result showed that this technique of AFSD showed a minimal titer reduction of ∼0.6 log in presence of trehalose-mannitol at a mass ratio of 7:3 thus advocating the further exploration of ASFD as an attractive alternative method over conventional freeze-drying processes providing similar biological preservative in a shorter time. **Table 1** provides a useful insight into recent *in vitro* and *in vivo* studies (2014 onwards), wherein different inhalation delivery methods (nebulizers, DPI, pMDI) have been used.

The major conclusions summarized from the studies of **Table 1** include that pulmonary delivery *via* nebulization and dry powder inhalation both represent a favorable and safe route (effective than other methods) for phage administration, enabling phage to reach the affected lung tissue and target respiratory pathogens. This is indicated by the significant reductions in lung bacterial counts as well as low inflammation seen in various animal studies. Secondly, although human studies using aerosolized, phage preparations are limited, results indicate good clinical tolerance with no side effects and complete resolution of infection over time. Pulmonary delivery may be used in combination with *i.v* administered phages or antibiotics. Combined administration of phage and antibiotics also showed higher reductions in bacterial burden and needs to be advocated further. The co-therapy mode (Phage and antibiotic) is an attractive approach over the traditional treatment protocols due to the proven synergistic antimicrobial effect (Torres-Barcelo et al., 2014; Kamal and Dennis, 2015; Oechslin et al., 2017]. The synergistic effect of phage PEV20 along with ciprofloxacin against the drug-resistant strain of *P. aeruginosa* administered *via* both air-jet and vibrating mesh nebulizers has been reported and studied in detail by Lin et al. (2018). Apart from the use of the nebulization method, Lin et al. (2019) also tested the SD-based inhalable powders of PEV20 and ciprofloxacin as dry powders for inhalation, which tend to show better patient compliance. Results showed that inhalable combination powder formulations of phage PEV20 and ciprofloxacin were stable and exhibited a strong synergistic antimicrobial killing effect against *P. aeruginosa* strains isolated from CF patients. Such findings advocate further research into the development of phage-antibiotic inhalable formulations for pulmonary delivery with improved and faster containment of infection. However, given the limited studies conducted to date on humans, more clinical research with a high sample size is required to understand the efficacy and safety of this approach. Finally, complete optimization studies need to be done for each phage (i.e which type of nebulization as well as the dry powder inhalation technique to be used, the choice of stabilizers, effect on phage morphology, stability and viability, lung deposition percentage, testing phage-antibiotic synergism, etc.) to ensure the greatest clinical benefits.

#### 2.3.3 Metered-Dose or Propellant Based Inhalation

Pressurized Metered-dose inhalation (pMDI) is based on a specifically designed device that delivers a minute and fixed amount of medication as a short burst of aerosolized form taken by the patient through their mouth (Ibrahim et al., 2015; Martin and Finlay, 2015). It contains three major parts, which include a) canister which holds the formulation b) metering valve, that allows a metered quantity of the formulation to be dispensed, and c) an actuator (or mouthpiece) allowing the patient to operate the device and it is attached to a nozzle which enables to spread the component in the mouth of the person using it. Metered-dose inhalers are mostly and more commonly used by asthmatics or people with COPD (Boyd, 1995; Brand et al., 2008). The drug formulation present in the canister is mixed with liquefied gas propellant and stabilizing chemicals. Such a metered-dose inhalation method offers the advantages of allowing the delivery of metered and specific amounts of medication, with no pre-drug preparation required and multi-dose capability available, while also being portable and comparatively inexpensive (Carrie, 2009; Javadzadeh and Yaqoubi, 2017). However, very little work has been reported on the use of this type of inhaler to deliver phage against RTIs. In a study by Hoe et al. (2014), phage suspension of two *myoviridae* phage (FKZ/D3 and KS4-M) was prepared using a reverse emulsion process with Tyloxapol as surfactant and filled into hydrofluoroalkane 134a pMDI canisters. The phages were actuated from the device and there was a negligible loss in titers, showing successful delivery to the lungs. Despite this, more dedicated studies on the different aspects of this inhaler for phage delivery are required. Moreover, one drawback is that just 10%–20% of the expelled dose reaches the lung (Liu et al., 2012; Chaturvedi and Solanki, 2013; Liang et al., 2020), which needs to be developed and improved.

#### 2.3.4 Soft–Mist Inhalation

A new class of propellant-free inhalers known as Soft Mist Inhalers (SMIs) have also been developed in recent years, also known as respimat inhalers. These inhalers release medication in a fine mist that comes out slowly. Hochrainer et al. (2005) showed that the velocity and spray duration of aerosols clouds released from SMI inhaler moved much slower and has a prolonged spray duration as well as compared to pMDIs and this will account for improved lung and reduced oropharyngeal deposition essential for moving outcome. SMIs come with a dose counter built-in, which enables us to see how many doses of medication are remaining and a lock itself system after the medication is all used up, but to date, there is limited data regarding this method. One study by Carrigy et al. (2017) compared the efficiency of phage delivery using vibrating mesh nebulizer, jet nebulizer, and soft mist inhalation (SMI) methods. The results showed that the SMI was able to deliver the mycophage D29 more quickly with high titers (~5 × 10^8^ PFU/actuation). There was a minimal titer reduction (0.6 log_10_ PFU/ml) and a higher lung delivery was achieved (3.2 × 10^6^ PFU/actuation of inhalable active phage). Similar to MDIs, this device again needs more exploration in phage delivery.

## 3 Advances in Delivery and Formulations-Inhaled Phage Therapy

### 3.1 Surface Acoustic Waves Nebulization and High-Frequency Acoustic Nebulization For Improved Pulmonary Delivery

The nebulization process and the hydrodynamic stress it generates (as in the case of ultrasonic nebulizer and cavitational process for aerosol formation) have been shown to have a detrimental effect on phage morphology and overall viability in past studies (Astudillo et al., 2018; Leung et al., 2018). One approach is the use of surface acoustic wave (SAW) nebulizers. SAWs operate at considerably higher (>10 MHz) frequencies than the ultrasonic nebulizers and essentially comprise surface waves and do not drive cavitation. In the absence of large cavitational pressures, high surface vibrational acceleration is produced and the acoustic energy produced causes the drop interface to rapidly destabilize and break up to form aerosol droplets containing the therapeutic molecule (Rajapaksa et al., 2014). The entire process occurs within such a short period that it is not sufficient to degrade biomolecules and thus represents a much gentler way of procuring aerosol particles (Qi et al., 2008; Collins et al., 2012). With its ability to generate aerosols within the 1–5 µm aerodynamic diameter range required for maximizing deep lung deposition (Qi et al., 2009), particularly in the smaller bronchioles that are common sites of pulmonary infection, SAW nebulization is an ideal and efficient platform for pulmonary administration of various biomolecules (Rajapaksa et al., 2014; Alhasan et al., 2016; Wang et al., 2016). However, one drawback of most nebulizers including SAW nebulizers is the long administration time taken for adequate dosing to reach deeper areas.

One approach of potential interest is the novel acoustic wave platform (HYDRA) for advanced levels for nebulization. HYDRA nebulizers exploit the combined effects of both bulk wave nebulization and surface waves i.e SAW nebulization enjoying an advantage for higher output and improved efficiency and efficacy with better preservation of molecular structure and function (Cortez-Jugo et al., 2015; Kwok et al., 2020).In a recent study by Marqus et al. (2020), the authors assessed the capability of this low-cost and portable hybrid surface and bulk acoustic wave platform (HYDRA) to nebulize a phage K and lytic enzyme (lysostaphin). Results showed that the HYDRA platform was able to produce monodispersed phage aerosol particles within a defined size range (1-5 μm) ideal to be delivered to the lower respiratory airways and deep pockets. There was a minimal loss in the phage viability (negligible titer loss of 0.1 log_10_ (PFU/ml) with a high viable respirable fraction (90%) reaching the active site. This indicates that the HYDRA nebulization process does not result in appreciable denaturation of phages or even proteins (as seen with lysostaphin results) preserving function and structure. This calls for further exploration of this novel HYDRA nebulization platform for improved delivery of mono-disperse aerosol down to the lower airways, especially targeting chronic deep-seated infections.

### 3.2 Electrospray for Controlled and Targeted Drug Delivery *via* Inhalation

Although nebulization remains the preferred method of drug delivery *via* the inhalation route, it suffers from common pitfalls. Nebulization typically generates contaminant particles in the ultrafine size range from dried solutes and biological fragments in the nebulizer suspension. These contaminants can mask the size distribution of virus particles that are of comparable size (Hogan et al., 2004; Hogan et al., 2005), reducing the overall efficacy of the process. Another drawback observed is that some portion of the nebulized solution may flow back to the nebulizer reservoir, and fraction will evaporate over time causing the solution to become more concentrated (Chen and John, 2001) and again changing the aerosolized particle size distribution function which is not desirable (Eninger et al., 2009). In addition, with these traditional inhalation techniques including nebulizers, DPIs, and pMDIs, high deposition efficiency is often a problem with less than 20% of the spray reaching the target area of the lungs as most of the drug particles get deposited in the upper airway. Moreover, they tend to produce more of a polydisperse type of particle with varying diameters. The bigger diameter particle tends to deposit in upper airways rather than reaching lungs with less than the actual administered dose (or phage titers) reaching the actual site for action thus decreasing the desired outcome (Tena and Clarà, 2012; Cheng, 2014). Electrospray (ES) or electrohydrodynamic atomization (EHDA) is a promising atomization process due to its ability to produce a spray with monodisperse droplet size. It is an atomization technique that uses electro-hydrodynamic forces to disperse a liquid into fine droplets thus forming micro and nano-sized mono-dispersed droplets of the same and uniform size (Jaworek, 2007; Ryan et al., 2012). With the use of the electrospray process, the production of a relatively uniform narrow aerosol size distribution is achievable. The aerosolized formulation produced is comparatively without aggregates and free of generated contaminants from dried solutes i.e a cleaner and stable preparation (Thomas et al., 2004). There have been few dedicated studies focusing on this aspect.

Jung et al. (2009) investigated the characteristics of airborne MS2 bacteriophage particles <30 nm in size, using a charge-reduced electrospray technique. For this, the suspension of phage was sprayed cone-jet mode using a specially designed electrospray system in a cone-jet mode. Results indicated that the electro-sprayed MS2 particles so formed showed excellent monodisperse size distribution, high stability, and uniformity which was not seen with nebulized particles. Thus, the authors reported the electrospray method being able to produce non-agglomerated particles, resulting in a narrow size range of uniform size. In another study reported by Eninger et al. (2009), the aerosolization of bacteriophage MS2 virions by nebulization and charge-reduced electrospray were compared during testing of three filter media. Results depicted that although both aerosolization methods generated culturable MS2 virions electrospray method produced an airborne concentration of phages that was 20-fold higher than the nebulizer. The electrospray produced cleaner, more stable, and higher viable phages in the aerosolized particles as compared to the classical nebulization process. The nebulized aerosol particle count was 2.8 times more variable than the electro-sprayed aerosol particle count. This indicates that the nebulizer produced a poly-disperse aerosol, unlike the electrospray protocol, which also produced a more desirable and relatively mono-disperse aerosol and a better way of filter testing the delivery method. These findings encourage exploration of this mode of generating aerosolized phages and the possible effect of the electrospray technique on the viability of phage titers.

### 3.3 Liposome Encapsulated Phage Preparation for Improved Pulmonary Delivery

Liposomes are one of the lipid-based nano-vesicles that self-assemble, forming lipid nano-spheres that act as an ideal drug delivery approach for encapsulating and protecting phages, showing bio-compatibility with various phage preparations (Singla et al., 2015; Chadha et al., 2017; Chhibber et al., 2018; Otero et al., 2019). Liposome-loaded phages are protected from outer stress such as the action of body fluids, enzymes, clearance from the reticuloendothelial system (RES), the action of neutralizing antibodies (Colom et al., 2015; Singla et al., 2016; Chhibber et al., 2018; Leung et al., 2018). They are also capable of undergoing conformational transitions as they mimic biological membranes and this allows them to reach and penetrate the deeper areas crossing the host tissue barriers. This is especially important in the case of penetrating the biofilm-affected areas. Liposome encapsulation may enable phages to gain access into the eukaryotic cell to target intracellular pathogens, as free phages have limited ability to penetrate eukaryotic cells (Nieth et al., 2015). The use of liposome encapsulation technology in the delivery of phages and various antibiotics has been successfully reported by recent studies against a range of pulmonary pathogens. Singla et al. (2015) reported the successful encapsulation of phage KPO1K2, specific for *K. pneumoniae in* cationic liposomes with high efficiency of 92% and significant structural and biological stability for nine weeks at 4°C and room temperature. The liposomal preparation was able to protect all tested mice from pneumonia-induced death even when the therapy was delayed by 3 days after induction of infection by *K. pneumoniae* with complete clearance of organisms from the lungs within 72 hours after treatment. Liposomal encapsulated phage treatment also led to a higher reduction in inflammatory cytokines levels. Although the result shows the enhanced persistence of encapsulated phages in lung tissue and higher therapeutic effect against pneumonia, the liposomal phage preparation was here given intra-peritoneal and not tested *via* the inhalation route.

The biggest advantage of inhaled antibacterial therapy would be its ability to target intracellular respiratory pathogens such as *M. tuberculosis*. While studying liposome-mediated intracellular delivery, Nieth et al. (2015) reported the successful encapsulation of mycobacteriophages in giant unilamellar liposomes (≥ 5 μm) by two different techniques i.e gel assisted GUV formation and inversion emulsion technique. These liposome-associated bacteriophages were able to enter THP-1 cultured eukaryotic cells significantly more efficiently than free bacteriophages and co-localize with early- and recycling endosomes. Similarly, in a recent study by Vladimirsky et al. (2019), macrophage cell culture (RAW 264-7-ATCC) was first infected for 24 hours with *M. tuberculosis* strain i.e H37RV MTB at a concentration of 10^7^ CFU/ml and then incubated with free phage and liposome-encapsulated phage D29 to study the decline in bacterial counts post 24 h of co-incubation. The results of counting of MTB colonies showed 62 colonies in control (no treatment), 17 ± 1 in free mycobacteriophage treated and only 7 MTB colonies in the liposomal mycobacteriophage treated, showing significantly high bactericidal effect with liposomal phage preparation. These results indicate new opportunities for treating mycobacterial infections.

Besides the above studies, no major studies have directly focused on the preparation of inhalable liposome-encapsulated phage formulations and their delivery through liquid or dry powder aerosolization and their efficacy testing, although inhalable liposome loaded antibiotics against respiratory pathogens have been accessed in many studies (Waters and Ratjen, 2014; Grifth et al., 2018; Bassetti et al., 2020). Liposome encapsulated phage delivery *via* aerosolization may be associated with its own challenges. Firstly, during liposome formation following the conventional thin-film hydration and extrusion method, phages are exposed to the heat used during hydration and high mechanical stress generated upon extrusion, which may account for significant losses and low encapsulation efficiency (Colom et al., 2015). Even with an improved method such as gel assisted formation followed by extrusion and inversed emulsion, the liposome that are formed are large in size ≥ 5 (Nieth et al., 2015), which is not ideal for pulmonary delivery, as most of them may fail to reach deeper lung areas. Secondly, the major challenge is the stability of liposome vesicles during the nebulization process. The shearing stress of the nebulization process to convert liposome dispersions into fine aerosol droplets may result in vesicle fragmentation and loss of the encapsulated phage. Vesicles may also undergo marked size reduction during jet nebulization, as reported by Saari et al. (1999). These physical changes highlight that applying a mild nebulization technology to minimize the process of fragmentation and shear degradation of lipid nanovesicles. The inclusion of stabilizers such as cholesterol or high-phase transition phospholipids in the liposome formulations has been shown to exhibit a protective effect (Elhissi et al., 2007; Clancy, 2013).

Keeping the challenges in mind, these lipid-based nanocarriers represent an ideal platform for successful encapsulation and pulmonary delivery of the sensitive phages, phage cocktails, and even phage endolysins, while maintaining their viability and infectivity intact and thus, more research and future studies are required to explore this direction.

### 3.4 Individualized Controlled Inhalation Technology: Integrated Software Control

ICI technology is one of the most promising novel approaches for the improvement of pulmonary aerosol deposition, offering higher drug targeting, reduced lung dose variability with unique integrated software control (Chandel et al., 2019; Longest et al., 2019). The AKITA^®^ technology is the most advanced ICI technology-based aerosol delivery technology as it controls the entire inhalation maneuver of the patient resulting in more precise drug targeting. This is accomplished by positive air pressure delivered by a computer-controlled processor, which is made to program as per the patient’s individual lung function data, which is tested prior to use (Fischer et al., 2009; Kesser and Geller, 2009; Tashkin, 2016).

AKITA^®^ works well with ultrasonic mesh nebulizers and the latest versions are fully compatible with vibrating mesh nebulizers, delivering as high as 99% of the filled dose nebulized into aerosol particles with Median mass aerodynamic diameter (MMAD) of < 4 μm (Kesser and Geller, 2009; Fischer et al., 2009). Such ICI-based technology is associated with clear advantages of minimal dose variability and maximum efficiency as this technology enables better control over aerosol flow rates, delivery volumes, dosing timings giving higher compliance to the treatment protocol. In a cross-over study on inhaled tobramycin done in healthy individuals (Brand et al., 2005), it was observed that individuals using conventional jet nebulizer system achieved lung deposition of a total of 40.78 mg with as high as 30% variation in total lung dose while AKITA^®^ system showed deposition of 42.81 mg with less than 11% as the dose variation seen.

The integrated software controls provided with these technologies further allow the physician to have more control over the therapy and when to change the controls as per the constant monitoring of parameters and observed adverse effects which are possible with such latest systems (Bennett, 2005; Ibrahim et al., 2015). For example, past studies have shown that lung deposition of lipopolysaccharide, an endotoxin if present in drug formulations leads to triggering of airway inflammation and adverse effects in patients with COPD, CF (Thorn and Rylander, 1998; Muhlebach and Noah, 2002). However, this can be well controlled and managed by the use of such ICT systems with advanced features such as constant scanning of information about nebulized drug dose, treatment time, adverse effects if any. This feature is particularly useful in the case of phage preparations wherein endotoxin may contaminate the formulation owing to gaps during high titer phage production and purification if any. On similar grounds, there is another technology i.e the I-Neb^®^, which consists of high-level software control integrated with mesh nebulizer as a single device (Geller and Kesser, 2010; Tashkin, 2016). This system works on either of the two modes i.e a tidal breathing mode and a targeted-inhalation mode. In tidal breathing mode, the device aerosolization process is adapted as per the patient’s tidal breathing pattern. However, in the targeted inhalation mode, a vibrating feedback system guides the patient towards an optimal breathing pattern to enhance and further improve the aerosol deposition and final efficacy (Zhou et al., 2014).

Many studies on these ICT-based nebulizers and phage therapy have not seen the light yet. However, in the case of personalized pulmonary phage therapy, such software integrated systems (with better control over dosing volumes, dosing times, aerosolization rates, higher physician monitoring) that are optimized as per individual patients’ needs and lung function will help to further enhance the success and outcome of phage treatment (with more phages dose reaching the lower and deeper lung pockets). This is especially important while treating patients with recurrent chronic bacterial infections.

## 4 Conclusion

Inhaled phage therapy has the potential to transform the prevention and treatment of bacterial respiratory infections, including those caused by antibiotic-resistant bacteria. The results of various studies advocate that inhaled phage therapy is a safe and potent antibacterial option with no reported adverse events. There is a long way to go before clinical approval of inhaled phage therapy. Robust randomized clinical trials, a deeper understanding of the pharmacological studies of the inhalable formulations, and further research on the stability of phage in various formulations need attention for moving this therapy closer to final approval and use. However, the use of inhaled phage therapy on compassionate grounds needs to be looked at as a priority. Despite the concerns outlined here, inhaled phage therapy holds strong potential and represents a new era of inhalable phages that act on multiple fronts to resolve respiratory infection working well even against drug-resistant strains.

## Author Contributions

Literature search, data extraction, writing-review, and final editing: XW, ZX, JZ, ZZ, CY, and YL. All authors have reviewed and approved the final version of the article, including the authorship list.

## Conflict of Interest

The authors declare that the research was conducted in the absence of any commercial or financial relationships that could be construed as a potential conflict of interest.

## Publisher’s Note

All claims expressed in this article are solely those of the authors and do not necessarily represent those of their affiliated organizations, or those of the publisher, the editors and the reviewers. Any product that may be evaluated in this article, or claim that may be made by its manufacturer, is not guaranteed or endorsed by the publisher.
